# Four-Year Cumulative Radiation Exposure in Patients Undergoing Computed Tomography Angiography for Suspected Pulmonary Embolism

**DOI:** 10.1155/2013/482403

**Published:** 2013-07-28

**Authors:** Edwin A. Takahashi, Hyo-Chun Yoon

**Affiliations:** ^1^John A. Burns School of Medicine at the University of Hawaii, 651 Ilalo Street Honolulu, HI 96813, USA; ^2^Kaiser Foundation Hospital, Diagnostic Imaging, 3288 Moanalua Road, Honolulu, HI 96819, USA

## Abstract

*Purpose.* The objective of this study was to determine the estimated effective radiation dose of pulmonary CT angiography (CTA) for suspected pulmonary embolism (PE) contributing to total medical radiation exposure over a 4-year period. *Materials and Methods.* This investigation retrospectively reviewed 300 patients who presented to the emergency department and received a pulmonary CTA scan for suspected PE. We evaluated these patients' electronic medical record to determine their estimated radiation exposure to CT scans during the following four years. Using DLP to *E* conversion coefficients, we calculated the cumulative effective radiation dose each subject received. *Results.* A total of 900 CT scans were reviewed in this study. Pulmonary CTA delivered an average effective radiation dose of 10.7 ± 2.5 mSv and accounted for approximately 65% of subjects' 4-year cumulative medical radiation dose. Only 6.3% of subjects had a positive acute PE according to their radiology report. *Conclusion.* Pulmonary CTA accounted for the majority of subjects' medically related effective radiation dose over a 4-year period. With only a minority of subjects having positive findings for acute PE, increased efforts should be made to clinically assess pretest probability before the consideration of imaging.

## 1. Introduction

Pulmonary embolism (PE) is a significant cause of morbidity and mortality. In the United States, PE affects nearly 600,000 individuals and may result in approximately 100,000 deaths per year [1]. Presently, computed tomography (CT) angiography (CTA) has largely replaced the ventilation/perfusion (V/Q) lung scan and catheter pulmonary angiography for the diagnosis of PE due to its accessibility, reliability, and noninvasiveness [2–5]. However, only a minority of CTA scans ordered for suspected PE yield positive findings [1, 5–7]. The PIOPED II multicenter trial has also shown that this test is not as sensitive or specific as previously reported [8].

 The health risks associated with medical imaging may increase as the cumulative radiation dose accumulates over a lifetime. Certain factors such as patient age, gender, and fractionation of radiation impact the potential injury from radiographic imaging. A CTA scan delivers an effective radiation dose of approximately 10 mSv [9]. Several controversial studies have attempted to estimate the cumulative carcinogenic risk from medical imaging. Exposure to ionizing radiation from CT has been estimated to be responsible for as many as 29,000 malignancies in the United States annually [10]. Brenner et al. report that a lifetime exposure of 50–100 mSv may increase the risk of cancer, while exposure exceeding 100 mSv is almost certainly linked to cancer and other medical problems [11]. Despite the debate over findings such as these, there remains evidence to suggest that we should be vigilant in the use of imaging that utilizes ionizing radiation which poses a risk, however small, for inducing cancer. 

At this time, there is limited data on the impact of ionizing radiation from pulmonary CTA on the cumulative effective radiation dose in patients. The aim of this study is to determine the effective radiation dose received by patients with suspected PE from CTA and compare it to the total effective radiation dose over a 4-year period following the initial PE study. 

## 2. Materials and Methods

### 2.1. Study Design and Cohort Selection

This retrospective cohort study was performed at a 265-bed urban health maintenance organization (HMO) hospital. Institutional review board approval was obtained for this Health Insurance Portability and Accountability Act-compliant study. Informed consent was waived for retrospective medical records review. The inclusion period for this study began in February 2006 when we began documenting estimated radiation dose on all CT studies within the PACS system and ended in November 2007. From the inclusion period, all effective radiation doses from CT were tracked for the following four years, which was the maximum timeframe in our medical records at the time of this study. 

Three hundred consecutive subjects who presented to the emergency department (ED) and received a pulmonary CTA for suspected PE during the inclusion period and for whom we had complete 4-year follow-up data following the pulmonary CTA were included in this study. Subjects were excluded prior to data collection if they were older than 70 years of age at the time of the exam. This cutoff in age was chosen because the risk of harm from radiation exposure decreases with increasing age [10]. Hence, we purposefully excluded those over 70 years of age due to the tapered implications of cumulative radiation in this less radiosensitive population. Additional subjects were excluded following data collection if they passed away within the 4-year period following their initial PE study or did not have continuous health insurance coverage through the HMO since this would inhibit the collection of comprehensive imaging datasets. 

### 2.2. Data Collection and Analysis

 Pulmonary CTA scans were performed in the craniocaudal direction from 2 cm above the aortic arch to the domes of the diaphragms using a multislice computed tomography unit (GE LightSpeed QX/i, General Electric Co., Milwaukee, WI, USA) with 4 detector arrays, 1.25 mm collimation, 120 kVp, 300 mA, and a pitch of 1.5, followed by 5 mm slices to image the lung apices and bases. A reduced kVp (80 or 100) was utilized in small or thin patients at the CT technologist discretion, but there were no formal weight or BMI based criteria. The CT protocol also used the standard automated dose modulation package (GE) available during the study period. Subjects were injected with 120 mL of Omnipaque 300 (GE) at a rate of 3 mL/s. 

Age, gender, radiation dose, pulmonary CTA results, and cancer history were obtained from medical records of all patients who underwent pulmonary CTA during the study period. Data analysis was performed using the Student' *t*-test for continuous variables and chi-square test for categorical variables. Statistical significance was defined as *P* ≤ 0.05. 

### 2.3. Effective Dose Estimation

Effective dose is a complex value that accounts for the amount of radiation exposed organs receive from radiographic imaging and each organ's sensitivity to carcinogenesis from this exposure. There are two available methods for calculating effective radiation dose (*E*). The first is to use organ and tissue dose coefficients derived from Monte Carlo simulations and International Commission on Radiological Protection specified tissue weighting factors. In this model, radiation dose index is estimated based on the summation of radiation exposure in individual organs. The second method involves a simplified model using dose-length product (DLP) to *E* conversion coefficients (*k*) reported by Shrimpton and coworkers from the European guidelines for multislice computed tomography [12]. This model has been shown to be reasonably robust and consistent for estimating the effective dose and was chosen for this study. Effective radiation dose in anatomical regions such as head, chest, and abdomen, rather than in specific organs, was calculated by multiplying DLP by *k*. For CT studies involving more than one anatomic region, the largest *k* value was selected to calculate the radiation dose.

## 3. Results

In order to accrue 300 subjects, we retrospectively examined the medical records of 514 subjects under the age of 70 years who received a CTA in the ED for suspected pulmonary embolism between February 2006 and November 2007. Forty of the 514 subjects (7.8%) had a positive PE officially read on pulmonary CTA. Of the 514 subjects, 145 patients were excluded because they did not maintain at least 4 years of continuous health insurance coverage from the time of CTA. Another 69 patients were excluded because they passed away within four years of their CTA. 

There was a significant difference in age between subjects who lost insurance coverage and those who stayed enrolled (44.4 versus 50.7 years old, resp.; *P* < 0.01). There was no significant difference in female to male ratio between those who lost insurance coverage and the 300 subjects included in this study (F : M ratio 79 : 66 versus 189 : 111, resp.; *P* = 0.09). The demographics for the initial 514 subjects as well as for the 300 living subjects with 4 years of continuous health coverage are given in Table 1.

A total of 900 CT studies were performed on these 300 subjects during the 4-year study period inclusive of the original CTA examinations. The mean age and standard deviation of the 300-patient cohort was 50.7 ± 13.4 years. There were 550 and 350 total CT studies performed on females and males, respectively, which averaged to 2.9 scans per female subject and 3.2 scans per male subject (*P* = 0.59). The number of CT scans taken by anatomic distribution is given in Table 2. The majority of CT scans were of the chest followed by the abdomen and pelvis.

 The mean cumulative 4-year effective radiation dose was 31.1 ± 40.5 mSv. A broad range of cumulative radiation doses were recorded among the subjects with a low dose of 7.4 mSv from a single CT scan to a high dose of 297.3 mSv from 12 CT scans. Pulmonary CTA accounted for an average of 10.7 ± 2.5 mSv. 

**Figure 1:**
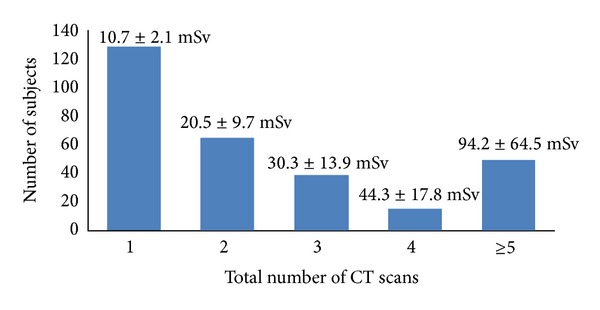
Number of CT scans received by subjects over four years inclusive of the initial pulmonary CTA for suspected PE. Mean effective radiation dose is displayed over each subject group.

The mean contribution of pulmonary CTA to the total 4-year radiation dose was 65.3 ± 35.1%. For 129 subjects, CTA was the only CT scan performed over the 4-year period. Only 4 out of these 129 subjects, or 3.1%, had an acute PE diagnosed by CTA.  Figure 1 shows the number of CT scans performed and average effective radiation dose subjects received over the 4-year study period. There was no gender difference in mean total radiation dose (females = 30.3 ± 37.0 mSv versus males = 32.6 ± 46.0; *P* = 0.65) or radiation dose from CTA alone (females = 10.5 ± 2.6 mSv versus males = 11.0 ± 2.2 mSv; *P* = 0.11).

Of the 300 subjects, 19 (6.3%) had an acute PE identified on CTA. The number of CTA studies performed and the number of positive PE increased by age group. In subjects ≤20 years of age, six CTA scans were performed with one PE identified. In subjects 21–50 years of age, 128 CTA scans were performed of which five PEs were identified. Lastly, in patients 51–70 years of age, 166 CTA scans were performed with 14 pulmonary emboli identified. 

Forty-seven subjects (15.7%) had a cumulative radiation dose >50 mSv. Of these subjects, 14 received greater than 100 mSv over four years with an average of 14 CT scans per person. The highest number of scans a subject received in this study was 24, which resulted in a cumulative effective dose of 164.3 mSv. 

Thirty-five subjects had previously diagnosed cancer and 17 subjects had a malignancy diagnosed within four years after their PE study. These subjects received significantly more mean cumulative radiation exposure than those who were cancer free (48.6 versus 28.4 mSv, resp.; *P* = 0.01). Of the 47 subjects in this study who received >50 mSv of radiation exposure, 13 had a cancer diagnosis either before or after receiving the index pulmonary CTA. This represents a significantly higher prevalence of cancer compared to the population with <50 mSv of cumulative radiation dose (*P* = 0.03). 

## 4. Discussion

The health risks associated with radiation exposure have been extensively studied. Epidemiologic data suggests that cancer risk is increased with acute effective radiation doses ranging from 10 to 50 mSv and for protracted doses between 50 and 100 mSv [11]. Roughly, 50% of the total radiation dose index from medical imaging comes from CT scans, which validates the attention these radiographic studies receive regarding patient radiation exposure [13]. Numerous studies have attempted to determine the cancer risk from pulmonary CTA. Cronin and colleagues report that there are approximately 150 excess cancer deaths per million people exposed to a single CT exam for PE [14]. A separate study by Smith-Bindman estimates that, among 20-year-old patients, there is one radiation-induced malignancy for every 330 females and 880 males who undergo CTA for suspected PE [15]. 

With a positive PE rate of <7%, our results suggest that pulmonary CTA was overutilized for suspected PE and exposed patients to a significant effective radiation dose. The effective dose from the initial pulmonary CTA scan accounted for an average of 65% of the cumulative effective radiation dose over the 4-year study period. The incidence rate of PE at our institution was lower than that reported in the literature of approximately 10–15% [2, 16]. This finding is likely a result of our exclusion of patients who passed away within the timeframe of this study.

 Pulmonary CTA was the only CT scan performed in 129 subjects over the 4-year period and delivered approximately 21% of the minimum radiation exposure estimated to increase the lifetime cancer risk. However, acute PE was diagnosed in only 3.1% (4/129) of these studies, which suggests that this cohort was of low risk and relatively healthy given that they did not receive further CT imaging in the 4-year study period. A meta-analysis investigating the prevalence of incidental, asymptomatic PE identified on CT scan concluded an overall weighted mean prevalence rate of 2.6% with ranges between 1.2% in outpatients and 4.0% in inpatients [17]. Another study by Storto et al. reported similar rates of incidental PE with 0.9% in outpatients and 4.0% in inpatients [18]. The rate of positive PE in our low risk subgroup, which fell within the range of incidental findings, implies that an effort should be made to stratify patients with suspected PE into lower and higher risk groups by referring physicians. 

Despite a consensus regarding CT overuse, imaging is still heavily ordered in the diagnostic workup for PE [2, 19, 20]. While this test is highly specific and a positive diagnosis of PE is typically accepted, the high incidence of negative exams suggests that CTA is used more for screening than diagnosis [7]. An assessment of pretest probability may help reduce the dependence on imaging in the ED. The Wells criteria have been shown to be reasonably accurate at classifying a patient's risk for PE [21]. Reported drawbacks for this screening tool include interobserver variability as well as variable adherence to using the Wells criteria in the clinical setting [2, 22]. The use of a quantitative serum D-dimer may also aid in establishing pretest probability. Multiple studies have demonstrated that a low serum D-dimer level has a strong negative predictive value [23–25]. Furthermore, its high sensitivity makes it a useful tool for ruling out PE in low to intermediate risk patients [6, 26]. Thus, the use of the Wells criteria in conjunction with the D-dimer assay may be able to reduce the overuse of CTA if they are used initially to assess pretest probability. Our institution does not mandate the use of pretest probability screening for PE prior to ordering imaging. However, establishing interdepartmental communication within the hospital may decrease the overuse of imaging. Further work to determine the rate of D-dimer and Wells criteria implementation in our ED will allow us to quantify the impact of these tools on pulmonary CTA ordering habits by ED physicians. 

Tracking cumulative radiation exposure is a growing concern in medicine. Shih et al. propose that tracking radiation and identifying trends related to CT radiation exposure is needed in order to detect inadvertent radiation overexposure and to better understand the risk of healthcare related radiation [27]. In this study, 145 of the 514 subjects (28%) whose medical records were initially examined lost continuous medical coverage with the HMO over the 4-year period following their initial CTA exam. This finding is concerning because the patients' record of radiographic imaging and thus their history of medically related radiation doses will be lost unless specifically requested by future healthcare providers. Furthermore, these excluded subjects were significantly younger than those who maintained continuous health insurance. Consequently, the health risks associated with lifetime radiation exposure will be difficult to evaluate, and the risk for overexposure in these subjects may be increased due to their younger age. 

The subjects in this study who were diagnosed with a malignancy before or within four years of their initial pulmonary CTA scan received significantly more cumulative radiation compared to subjects who were cancer free. The prevalence of cancer was also higher in those who received >50 mSv of cumulative radiation dose. These results can be rationalized by the frequent use of medical imaging in the diagnosis and management of malignancy. 

## 5. Limitations

Although PE is a significant cause of morbidity and mortality in the inpatient setting, this study only examined data from patients who presented to the ED. Management of patients in other settings may vary from the ED and the rates at which CTA is ordered for diagnostic assessment likely differ. The calculations of effective dose are estimations using DLP to *E* conversion coefficients, which are approximations for relative biologic risk. These coefficient values are based on data averaged over many different scanners and thus are not specific for the scanner used at our facility. However, the purpose of *k* is to be a universal coefficient that allows comparison across different CT studies and imaging tests. Another inherent limitation in this study pertains to the exclusion of patients who either died or lost insurance during the study period. This measure was taken to ensure accurate and comprehensive imaging history records. However, in doing so, a considerable portion of the initial subjects was excluded. 

Finally, the implementation of more advanced methods to reduce radiation dose from CT scans such as the adaptive statistical iterative reconstruction (ASIR, GE Co., Milwaukee WI, USA) we now have on our institution's CT scanners will reduce the overall radiation dose to patients even if the overuse of CT imaging continues. However, it is important that we continue to educate both referring physicians and patients about the need to avoid unnecessary radiation whenever possible by the application of appropriate clinical diagnostic algorithms.

## 6. Summary

In conclusion, pulmonary CTA accounted for 65.3% of total effective CT radiation doses in subjects with suspected PE over a 4-year period with only 6.3% of subjects having positive PE findings. Although pulmonary CTA is a fast and widely available tool for diagnosing PE, the high rate of negative scans in this study, as well as in the literature, suggests that more can be done to reduce potentially unnecessary imaging. We hope this study will stress the need for establishing diagnostic protocols in the ED. 

## Figures and Tables

**Table 1:** Demographics of subjects.

Demographic data from the original 514 medical records examined	
Mean age	50.1 ± 14.0 years
Age range	16–70 years old
Male/female ratio	200/314
Number of PEs identified by pulmonary CTA	40 (7.8%)

Demographic data from 300-subject cohort	

Mean age	51.2 ± 13.2 years
Age range	16–70 years old
Male/female ratio	108/182
Mean cumulative radiation dose	31.1 ± 40.5 mSv
Number of PEs identified by pulmonary CTA	19 (6.3%)

**Table 2:** CT scans performed over the 4-year period including the initial CTA for PE evaluation.

Body region	Number of scans
Head	164
Head and neck	4
Neck	22
Neck and chest	2
Chest	468
Chest and abdomen	8
Abdomen	34
Pelvis	5
Abdomen and pelvis	175
Chest, abdomen, and pelvis	18

Total	900
